# Bioinformatic prediction of key genes involved in pro-chondrogenic effect of fragmentated cartilage transplantation

**DOI:** 10.1038/s41598-025-05961-7

**Published:** 2025-07-01

**Authors:** Zhu Dai, Yong-Hui Jiang, Ying Liao, Lin He, Wen-Ji Yang, Jiang-Hua Liu

**Affiliations:** 1 Speciality of Sports Medcine, Department of Orthopaedics, the Fifth Affiliated Hospital, Sun Yat-sen University, Zhuhai, 519000 Guangdong China; 2 The First Affiliated Hospital, Department of Orthopaedics, Hengyang Medical School, University of South China, Hengyang, 421001 Hunan China; 3 The First Affiliated Hospital, Department of Intensive Care Unit, Hengyang Medical School, University of South China, Hengyang, 421001 Hunan China

**Keywords:** Cartilage fragmentation, Inflammation, DEGs of RNA sequencing, Trauma, Experimental models of disease

## Abstract

Minced cartilage transplantation is thought to promote cartilage repair. However, the underlying mechanisms remain less well understood. In this study, we established a rat osteochondral defect model to evaluate fragment size-dependent repair efficacy, and tried to explore the mechanisms preliminarily. Herein, rats with cartilage defect were randomly divided into 3 groups. Small allogeneic cartilage fragments with fibrin glue, chunk allogeneic cartilage fragments with fibrin glue, and only fibrin glue were used to treat cartilage defects in each group, respectively. The results showed that the minced cartilage fragments had significantly improved outcomes in promoting cartilage repairing compared to chunk cartilage fragments and only fibrin glue. Notably, particulated cartilage transplantation-treat cartilage lesion had elevated inflammation. Following RNA-seq analysis on cartilage fragments and cartilage chunk identified 75 differentially expressed genes (DEGs), which include 70 up-regulated DEGs and 5 down-regulated DEGs in cartilage fragment group (CFG). Further GO enrichment and KEGG pathway analysis showed that the up-regulated DEGs in CFG were mainly involved in inflammation, cell proliferation and migration. We also found that the down-regulated DEGs in CFG had negative relationship with cell migration, proliferation and inflammation. This study suggest that cartilage fragmentation enhances repair efficacy compared to chunk cartilage transplantation, and the mechanism of pro-chondrogenic effect may be related to inflammatory stimulation.

**Supplementary Information:**

The online version contains supplementary material available at 10.1038/s41598-025-05961-7.

Articular cartilage serves a critical biomechanical role in load-bearing, shock absorption and joint lubricating during sports. Among synovial joints, the knee exhibits particularly high susceptibility to cartilage defects due to its complex loading patterns^1,2^. It was reported that 5 − 10% patients were found to have full-thickness cartilage lesions during knee arthroscopic surgery^3^. The tissue’s intrinsic limitations, including avascularity, aneural architecture, low chondrocyte density, and dense extracellular matrix (ECM), severely constrain its self-repair capacity following injury^4^. Severe or unrepaired cartilage damage frequently progress to pain, joint dysfunction, deformity, and eventually osteoarthritis, which bring huge economic and medical burden to individuals and society^5^. While current clinical interventions range from microfracture to autologous chondrocyte implantation and tissue-engineered constructs, their widespread application remains hampered by inconsistent efficacy and prohibitive costs^6^. Therefore, the unmet clinical need underscores the imperative for novel regenerative strategies.

Among existing therapeutic approaches, osteochondral grafting demonstrates superior mid- to long-term outcomes^7^. Building upon this concept, minced cartilage transplantation has evolved as a promising alternative since its conceptualization by Albrect et al. in 1983^8,9^. Following investigation demonstrated that the chondrocytes embedded in extracellular matrix can migrate to the edge of cartilage fragments^10^. Subsequently clinical implementations, including CAIS (Cartilage Autograft Implantation System), DeNovo Natural Tissue (NT; ISTO, St. Louis, MO) and PJAC (particulated juvenile allograft cartilage), have demonstrated partial success^11^. However, most of clinical studies are limited to observe the relief of patients’ symptoms and the morphological characteristics of repaired cartilage. In terms of mechanism studies, our group previously found that the pro-migratory effect of chondrocytes induced by cartilage fragmentation^12^ was mediated by expression of MMP14 (matrix metalloproteinase 14, also known as membrane-type 1 matrix metalloproteinase)^13^. However, the comprehensive molecular mechanism governing this process warrants further elucidation.

The accelerated evolution of genomic sequencing technologies has equipped researchers with multifaceted tools to elucidate genetic landscapes across biological specimens. While contemporary single-cell approaches resolve transcriptional heterogeneity among cellular subtypes^14^the chondrocyte-centric architecture of articular cartilage^15,16^ renders bulk transcriptomic profiling via RNA sequencing (RNA-seq) indispensable for characterizing inter-sample molecular divergence^17^. To the best of our knowledge, transcriptomic perturbations associated with cartilage fragmentation remain systematically unexplored. To address this knowledge gap, we implemented RNA sequencing to systematically map fragmentation-induced transcriptional dynamics in chondrocyte populations.

A well-orchestrated inflammatory response is supposed to be a mediator for optimal tissue regeneration^18^. Previous study showed that noninfectious stimuli-induced immune reaction can trigger the recruitment of mesenchymal stem cells (MSCs) to injured area and accelerate the process of cartilage repair^19^. Mechanistically, proteolytic matrix remodeling during inflammatory phases enables transition of quiescent chondrocytes to activated states^20^with these mobilized cells demonstrating migratory capacity toward defect peripheries where they orchestrate ECM reconstitution via coordinated secretion of collagen-proteoglycan complexes and glycoprotein deposition^16^a self-limiting repair program harnessing inflammatory signaling for structural rehabilitation.

In this study, we establish a rat osteochondral defect model to compare reparative outcomes across cartilage fragment sizes. RNA sequencing of minced cartilage versus chunk cartilage revealed pronounced upregulation of pro-inflammatory pathways in fragmented specimens, corroborated histologically by elevated inflammatory mediator expression. This study aimed to demonstrate the therapeutic role of minced cartilage transplantation in cartilage repair and to uncover the underlying mechanism elementarily.

## Results

### Fragmentation improved repairing effect of cartilage implantation on osteochondral defect

**Fig. 1:**
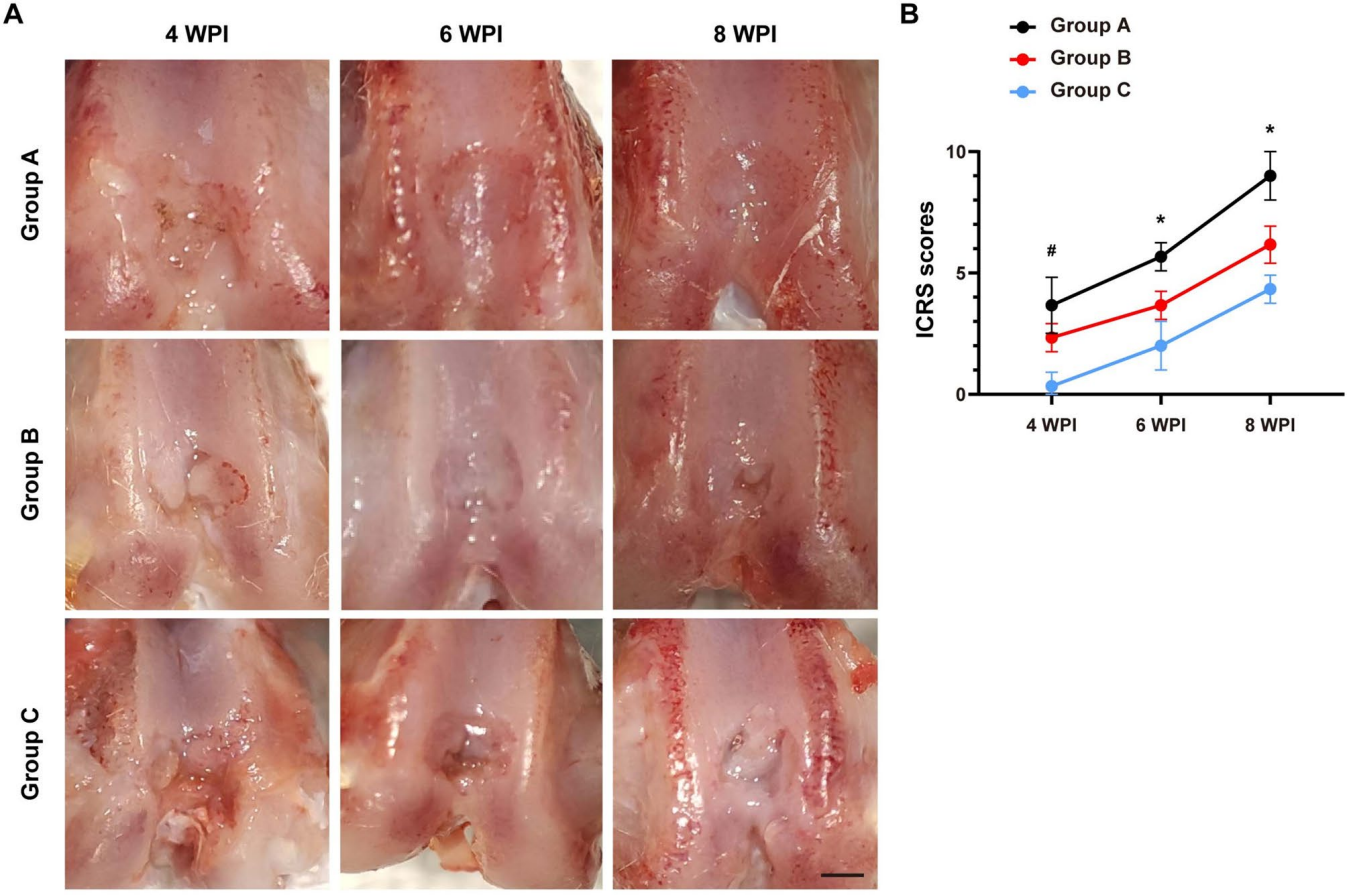
Appearance of knee joint specimens. **(A)** Representative images of general appearance of each group at 4, 6 and 8 WPI. **(B)** Quantitative analyses of ICRS scores of each group at 4, 6 and 8 WPI. *n* = 3. Rats in Group A received minced cartilage (mixed with fibrin glue) transplantation, rats in Group B were treated with chunk cartilage (mixed with fibrin glue) implantation, and cartilage lesions of Group C were only filled with fibrin glue. ^*^P < 0.05, Group A versus Group B. ^#^*P* < 0.05, Group B versus Group C. Data are mean ± SD. Two-way ANOVA with Turkey post-hoc test was used to perform multiple-group comparisons. Scale bar = 1 mm.

Firstly, macroscopic evaluation confirmed secure graft integration within osteochondral lesions across all groups, with preserved synovial architecture and absence of infection. As shown in Fig. 1, the osteochondral defects in all 3 groups were gradually decreased from 4 weeks post injury (WPI) to 8 WPI. Notably, the Group A (treated with minced cartilage and fibrin glue) demonstrated superior regenerative outcomes than other 2 groups at all timepoints (4, 6 and 8 WPI). Additionally, hyaline-like repaired tissue can be observed in defect area of Group A at 8 WPI, while the Group B (treated with chunk cartilage and fibrin glue) and Group C (treated with fibrin glue, served as control group) exhibited diminished regenerative capacity relative to Group A (Fig. 1A). International Cartilage Repair Society (ICRS) macroscopic evaluation scores quantitatively showed that the Group A had higher score than Group B, and Group B exhibited stronger cartilage-repairing effect that Group C (Fig. 1B). These data indicate that the fragmentation improves effects of cartilage implantation on cartilage defect.

**Fig. 2:**
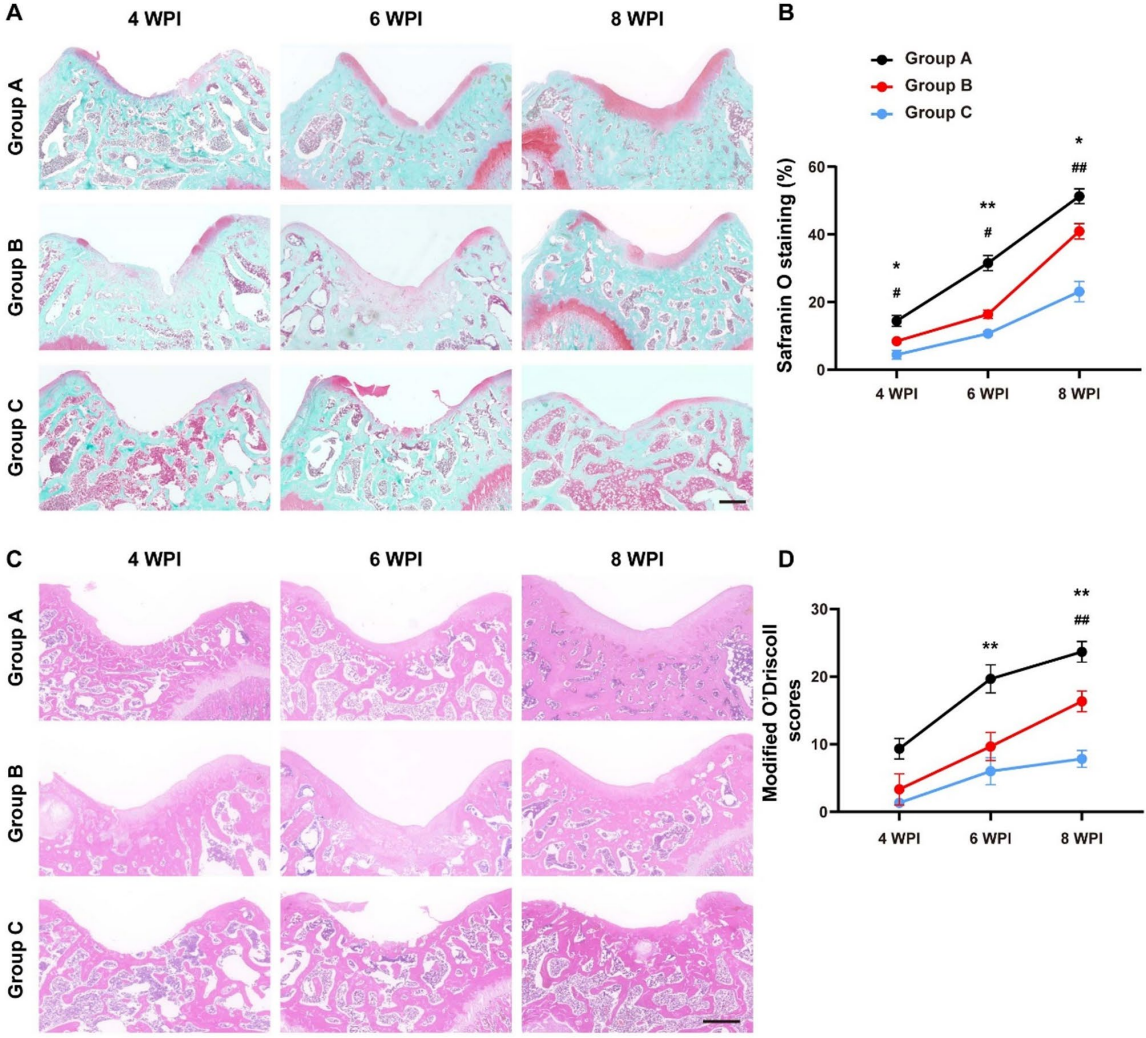
Histological evaluation of knee joint sections. **(A)** Representative safranin O/fast green staining images of each group at 4, 6 and 8 WPI. **(B)** Quantitative analyses of the safranin O positive-stained area of each group at 4, 6 and 8 WPI. **(C)** Representative HE staining images of each group at 4, 6 and 8 WPI. **(D)** Quantitative analyses of modified O`Driscoll scores of each group at 4, 6 and 8 WPI. *n* = 3. Rats in Group A received minced cartilage (mixed with fibrin glue) transplantation, rats in Group B were treated with chunk cartilage (mixed with fibrin glue) implantation, and cartilage lesions of Group C were only filled with fibrin glue. ^*^P < 0.05, ^**^P < 0.01, Group A versus Group B. ^#^*P* < 0.05, ^##^*P* < 0.01, Group B versus Group C. Data are mean ± SD. Two-way ANOVA with Turkey post-hoc test was used to perform multiple-group comparisons. Scale bar = 500 μm.

Next, we performed histological analyses to further evaluate the chondrogenic effects of 3 groups. It was found that Group A had greater neocartilage formation than that other groups, as evidenced by safranin O/fast green staining. At 8 WPI, the defect in Group A was basically healed and the repaired tissue in defect was highly similar to surrounding cartilage (Fig. 2A-B). HE staining showed that the minced cartilage transplantation (Group A) significantly accelerated cartilage defect repair and reduced articular surface lesion, as compared to chunk cartilage implantation (Group B) (Fig. 2C). In addition, the modified O’Driscoll scores, which were based on HE and safranin O/fast green staining images, demonstrated that the cartilage repair effect was obviously boosted by transplanted-cartilage fragmentation at all time points (Fig. 2D). These data, when taken together, suggested that minced cartilage transplantation had potential in promoting chondrogenesis of cartilage defect.

**Fig. 3:**
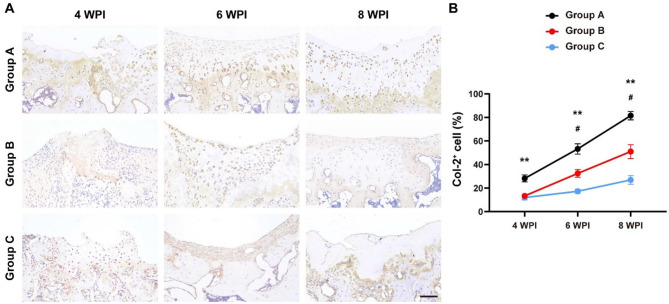
Col-2 immunohistochemical staining. **(A)** Representative Col-2 staining images of each group at 4, 6 and 8 WPI. **(B)** Quantitative analyses of the Col-2 positive-stained area of each group at 4, 6 and 8 WPI. *n* = 6. Rats in Group A received minced cartilage (mixed with fibrin glue) transplantation, rats in Group B were treated with chunk cartilage (mixed with fibrin glue) implantation, and cartilage lesions of Group C were only filled with fibrin glue. ^***^P < 0.001, Group A versus Group B. ^###^*P* < 0.001, Group B versus Group C. Data are mean ± SD. Two-way ANOVA with Turkey post-hoc test was used to perform multiple-group comparisons. Scale bar = 100 μm.

We also detected the expression of collagen type II (Col-2), a classic marker of chondrogenic differentiation^21^via immunohistochemical assays. As shown in Fig. 3, Col2-positive cells can be found in superficial, intermediate and deep layers, and the Col-2 staining intensities of Group A were much greater than that of Group B at 4, 6, and 8 WPI, while the cartilage formation was still enhanced by chunk cartilage fragments transplantation, as evidenced by the comparison between group B and group C. These results suggest that the cartilage transplantation can improve chondroid matrix formation and the pro-chondrogenic effect can be further promoted by fragmentation of implanted cartilage.

**Fig. 4:**
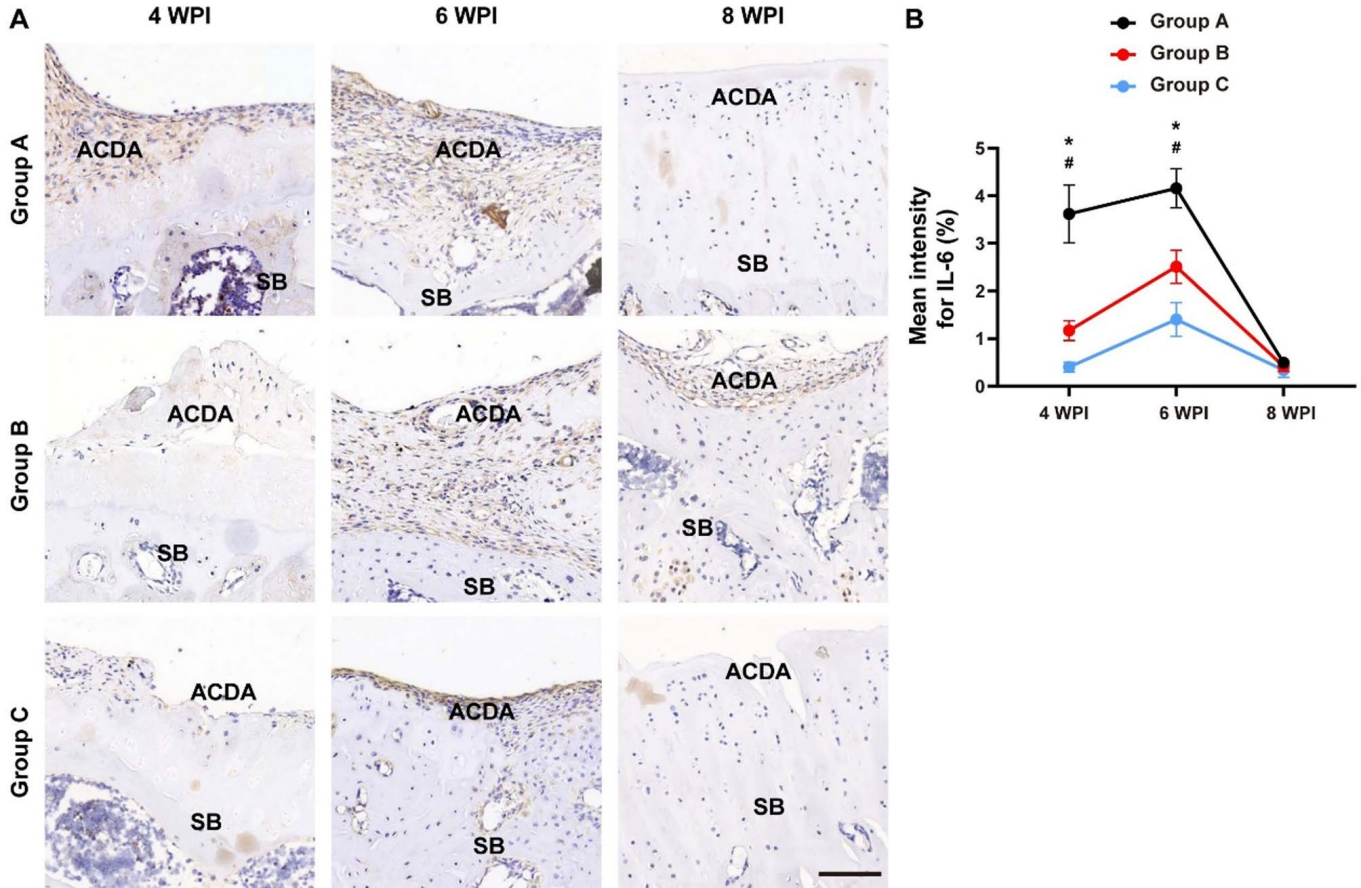
IL-6 immunohistochemical staining. **(A)** Representative IL-6 staining images of each group at 4, 6 and 8 WPI. **(B)** Quantitative analyses of the IL-6 positive-stained area of each group at 4, 6 and 8 WPI. *n* = 6. ACDA: articular cartilage defects area; SB: subchondral bone. Rats in Group A received minced cartilage (mixed with fibrin glue) transplantation, rats in Group B were treated with chunk cartilage (mixed with fibrin glue) implantation, and cartilage lesions of Group C were only filled with fibrin glue. ^*^P < 0.05, Group A versus Group B. ^#^*P* < 0.05, Group B versus Group C. Data are mean ± SD. Two-way ANOVA with Turkey post-hoc test was used to perform multiple-group comparisons. Scale bar = 100 μm.

Since it is widely accepted that inflammation plays an important role in cartilage regeneration^19,22^we detected the inflammatory factor expression in 3 groups. Unexpectedly, we found that the expression of IL-6, a pro-inflammatory cytokine, showed a trend of increase at 4 WPI and was obviously elevated by cartilage fragmentation at mid-stage (6 WPI) (Fig. 4). Specifically, the articular cartilage defects area (ACDA) of rats that received minced cartilage transplantation (Group A) showed much greater IL-6 staining intensity than that of rats treated with chunk cartilage (Group B) at 6 WPI, while the Group A and Group B exhibited similar inflammation level at 8 WPI. In addition, the rats only administrated with fibrin glue (Group C) had low inflammation level throughout the whole experiment. The above results suggest that a certain degree of inflammation can probably augment pro-chondrogenic effect of cartilage transplantation.

### Fragmentation altered the ‌‌transcriptomic profile of implanted cartilage

**Fig. 5:**
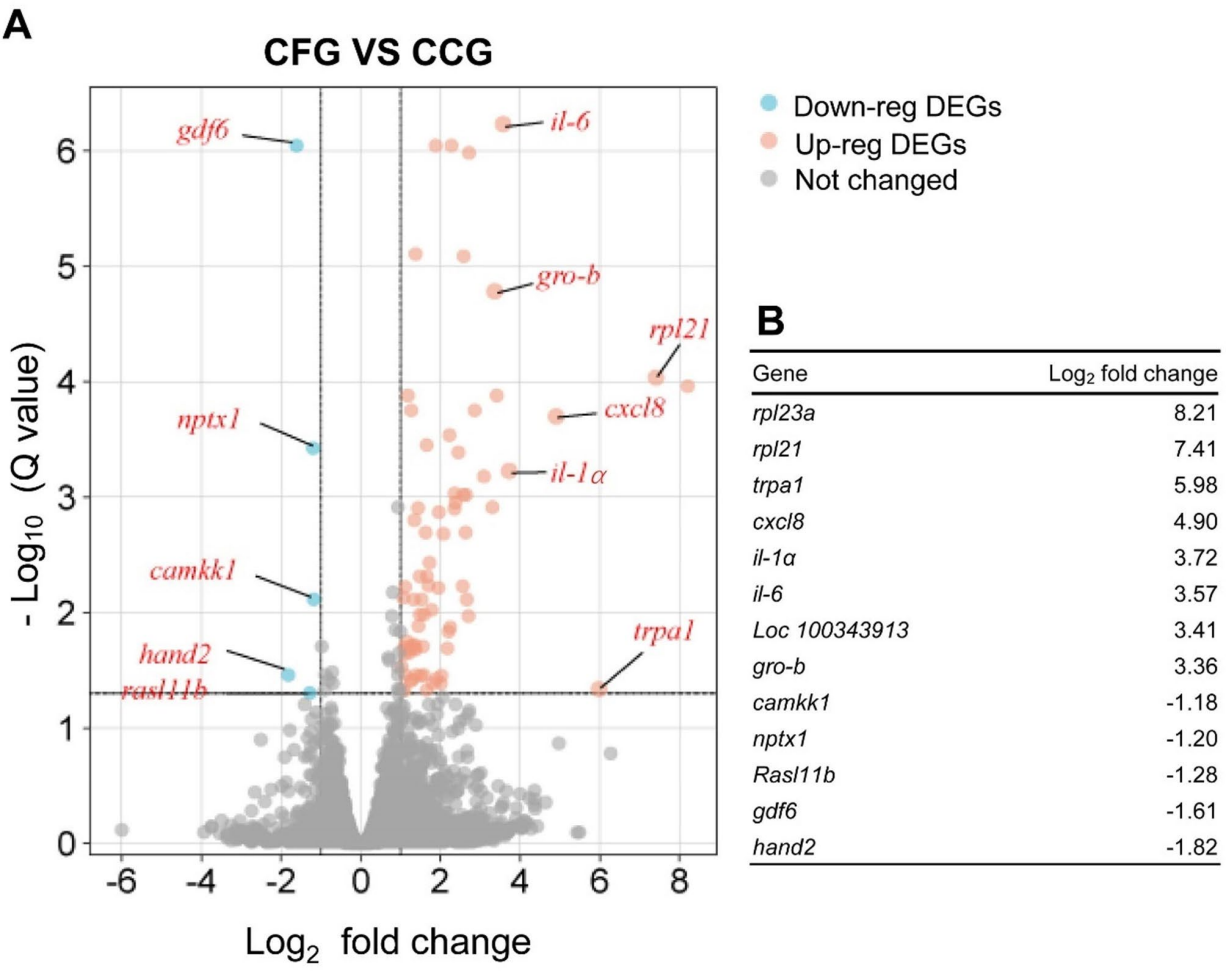
Volcano map of DEGs. **(A)** The X-axis is log2 scale of gene expression fold change, representing the alteration of gene expression in CFG relative to that in CCG. Y-axis is -log_10_ scale of Q value (adjusted *P* value), indicating the significant level of gene expression difference. The horizontal dotted line corresponded to 1-fold up and down, and the vertical dotted line represented a Q value of 0.05. The red points in the plot represented up-regulated DEGs in CFG group (Up-reg DEGs) and the blue points in the plot represented down-regulated DEGs in CFG group (Down-reg DEGs). **(B)** Top 8 up-regulated genes and top 5 down-regulated genes in CFG are shown.

**Fig. 6:**
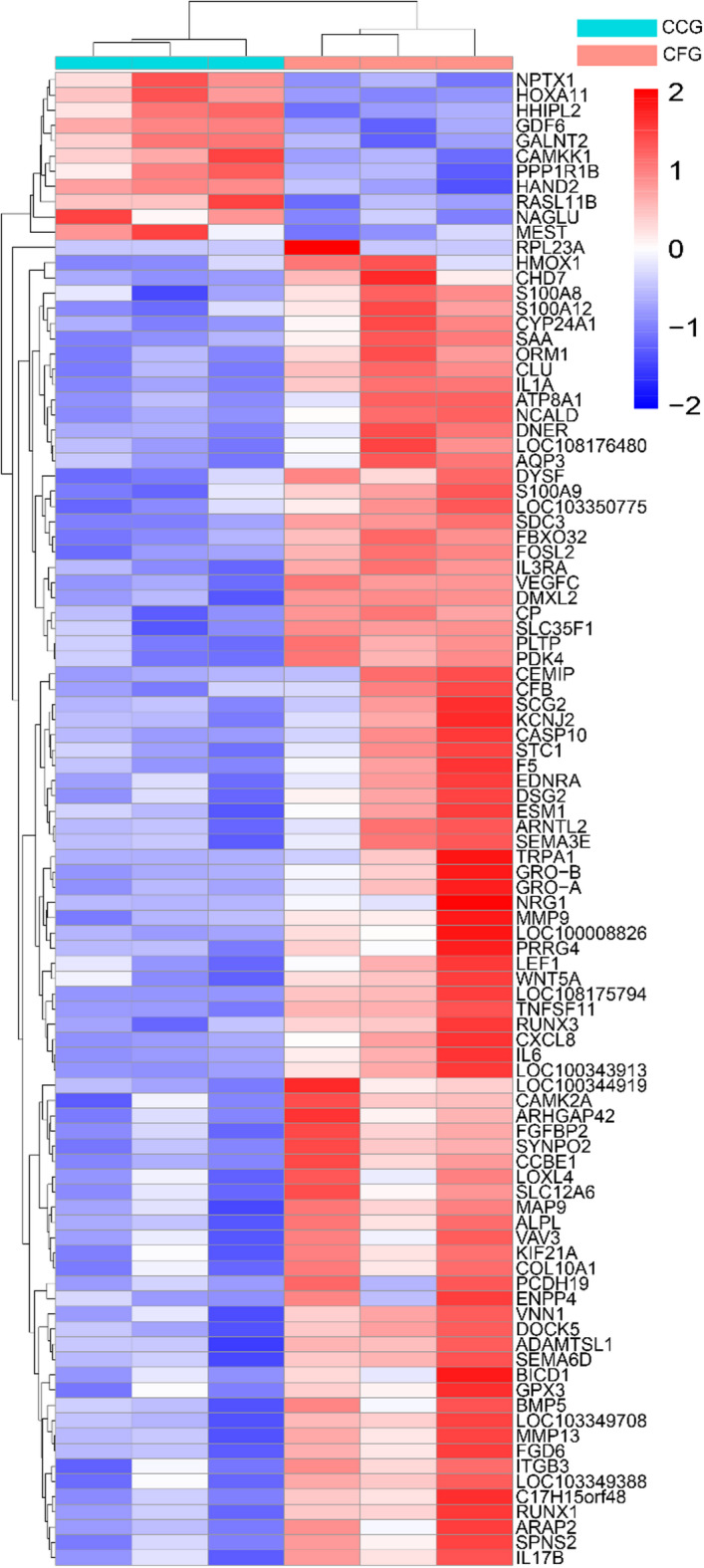
Heat map of DEGs. Expression profiles of the differential genes are scaled within each row. The highest expression value corresponds to bright red and the lowest to bright bule.

Given the observed correlation between transient inflammation and enhanced chondrogenesis in micronized grafts, we hypothesize that the increased inflammation may, at least to some extent, lead to a boosted chondrogenic response. In order to interrogate fragmentation-induced transcriptional reprogramming, and to explore the potential mechanism that mediate the pro-chondrogenic effects of cartilage fragmentation, RNA sequencing was performed to detect the mRNA expression profiles in 3 specimens of cartilage fragment group (CFG) and 3 specimens of cartilage chunk group (CCG). Totally, there were 18,977 genes identified in CFG and CCG, among which 75 differentially expressed genes (DEGs, Q value < 0.05; Log_2_ Fold Change ≥ 1) were obtained (Supplementary materials). As shown in Figs. 5A and 70 DEGs were up-regulated and 5 DEGs were down-regulated in cartilage fragment group (CFG), as compared to that in cartilage chunk group (CCG). The top 8 up-regulated DEGs and 5 down-regulated DEGs in CFG were listed in Fig. 5B. Among the top 8 up-regulated genes, we surprisingly found that *rpl21*, *trpa1*, *il-8*, *il-1α*, *il-6* and *gro-b* (C-X-C motif chemokine ligand 2, CXCL2/IL-2) were reported to involve in immunoreactions and exert pro-inflammatory effects^23–26^. Additionally, a hierarchical cluster analysis-based heat map was used to illustrate the total RNA expression of each sample, and significant differences between CFG and CCG were identified (Fig. 6).

**Fig. 7:**
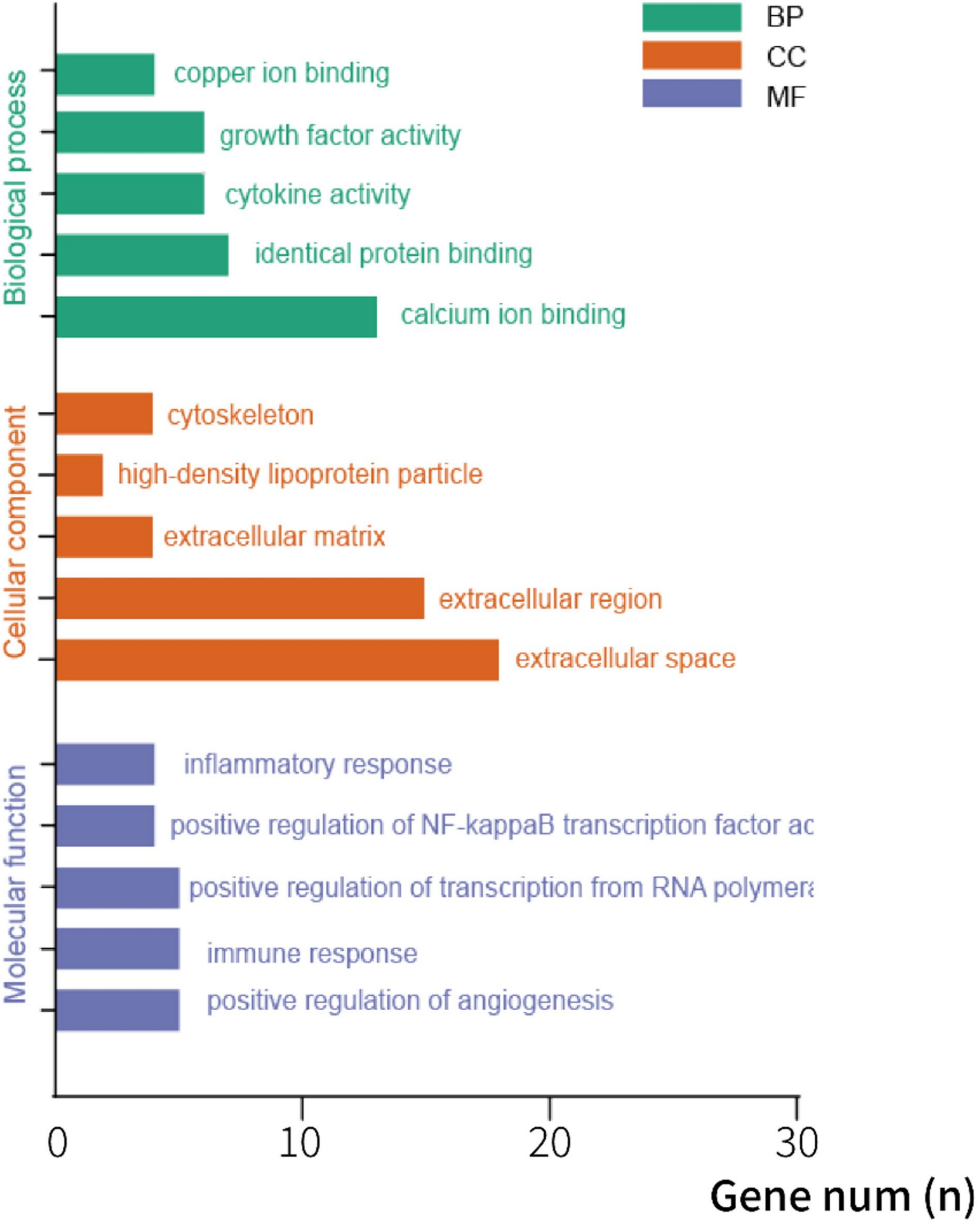
The gene ontology (GO) analysis of up-regulated DEGs in CFG. The Y-axis represents the GO terms significantly enriched in the categories of cellular component, molecular function, and biological process. The X-axis represents the number of mapped genes in each category.

GO enrichment analysis was carried out to further understand the biological functions of the chopped cartilage implants. We analyzed biological process (BP), cell component (CC) and molecular function (MF) of the up-regulated DEGs in CFG. As shown in Fig. 7, BP enrichment revealed that the up-regulated DEGs in CFG were mainly involved in calcium ion binding, identical protein binding, cytokine activity, growth factor activity and copper ion binding. The CC of differentially expressed genes mainly included in extracellular space, extracellular region, extracellular matrix, high-density lipoprotein particle and cytoskeleton. Of note, MF analysis showed that the functions of up-regulated DEGs are associated with positive regulation of angiogenesis, immune response, positive regulation of transcription from RNA polymerase II promoter, positive regulation of NF-kappa B transcription factor activity and inflammatory response (Fig. 7).

**Fig. 8:**
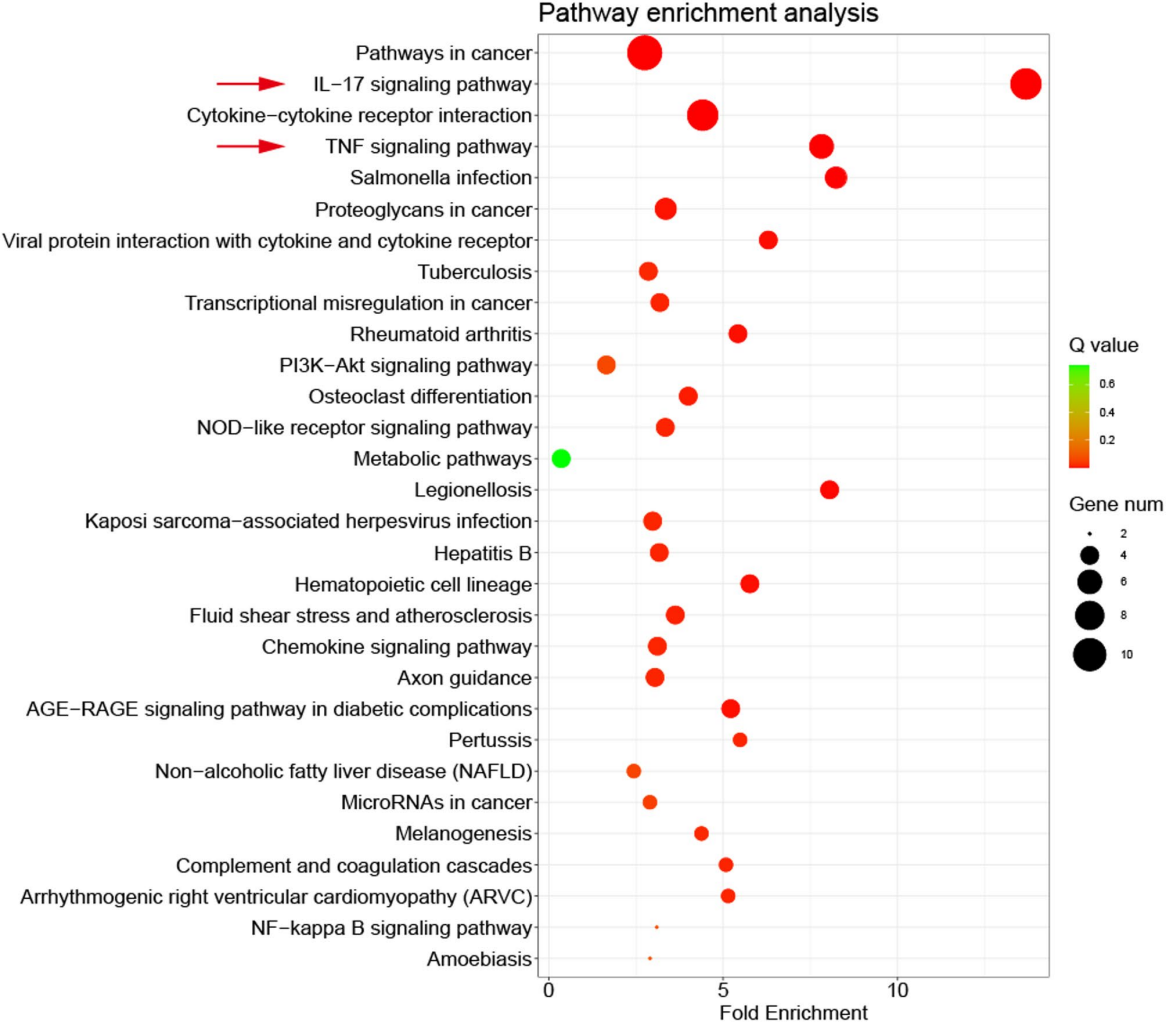
Scatter plot of enriched KEGG pathways of differential gene. The horizontal coordinate represents the fold enrichment, and the vertical coordinate represents the enriched pathway. The color and size of the dots represent the range of the Q values and the number of DEGs mapped to a certain pathway, respectively.

To study the possible functional pathways of up-regulated DEGs in cartilage fragment group (CFG), we carried out the KEGG (kyoto encyclopedia of genes and genomes gene ontology) pathway enrichment analyses^27^. As shown in Fig. 8, KEGG analysis results indicate that the up-regulated genes in CFG were primarily involved in IL-17 signaling pathway, TNF signaling pathway, and some microbial-like reactions (such as salmonella infection, viral protein interaction and tuberculosis). This systems-level interrogation aligns with transcriptomic (Figs. 5 and 6) and histological (Fig. 4) evidence collectively suggest that the fragmentation obviously promoted the inflammation level in cartilage.

## Discussion

Over the past decades, cartilage fragmentation has received increasing scientific interest since the chondrocytes in fragmented cartilage can migrate to the edge of fragment and promote cartilage formation^10^. However, the underlying mechanism remains unclear. In this study, we established a knee joint osteochondral injury animal model and found that the transplantation of cartilage fragments can effectively promote repair of cartilage defect, where newly formed hyaline-like and Col-2-rich repaired tissue. While in Group B (treated with chunk cartilage), only cartilage-like tissue was detected in cartilage lesion, especially at 6 WPI (Fig. 2A). These structures exhibited markedly reduced staining intensity compared to native cartilage, indicating diminished glycosaminoglycan (GAGs) content. GAGs, including chondroitin sulfate, and keratan sulfate, are critical components of functional cartilage matrix and correlate strongly with chondrogenesis. We speculated that this phenomenon was probably caused by insufficient regenerative capacity of the implanted chunk cartilage. Of note, cartilage fragments-repaired area showed increased inflammation, which prompted us to speculate that the highly-expressed inflammatory response may benefit the pro-chondrogenic effect of minced cartilage. The following RNA-seq analysis revealed a predominant enrichment of pro-inflammatory functional annotations among the top 8 up-regulated DEGs in the CFG (cartilage fragment group). This study provides an effective therapeutic method for cartilage lesion treatment and broadens our understanding in mechanism by which minced cartilage transplantation improves chondral defect repair.

Usually, inflammation is considered to be responsible for cartilage catabolism and can lead to a variety of chronic articular disease, such as osteoarthritis and rheumatoid arthritis^28^. However, like other tissue regeneration^18^moderate inflammation is also required by acute cartilage injury repair. Notably, the underlying mechanisms differ between vascularized tissues and avascular tissues. In former, injury-initiated inflammation and hemorrhage can transport immune cells (e.g., neutrophils, monocytes and macrophages) to wounded area to remove tissue debris and then recruit stem cells through blood stream to repair tissue damage^18^. For cartilage, chondrocytes normally remain quiescent in matrix. Once injury happens, augmented inflammation can induce degradation of cartilage extracellular matrix (ECM), and activated chondrocytes are released to produce ECM to regenerate cartilage^16^. In this study, transcriptomic evidence of grafts (cartilage fragments and cartilage chunk) showed that the fragmentation (injury of graft cartilage) can enhance immune response, suggesting that the high level of inflammation in minced cartilage-treated rat is probably derived from implant. According to these results, we supposed that the elevated inflammation level of minced cartilage probably leads to the degradation of ECM and chondrocytes secretion, thereby improving cartilage defect repair. Of note, the synovium also secretes inflammatory factors in response to trauma, which may modulate cartilage defect repair. The interaction between synovial activity and cartilage regeneration merits further investigation.

In top 8 up-regulated DEGs in cartilage fragments, *rpl21*, *trpa1*, *il-8*, *il-1α*, *il-6* and *gro-b* were considered as pro-inflammatory genes. By reviewing literature, we found these genes exert some other effects that also can promote cartilage repairing. For example, *rpl21*, *trpa1*, *il-1α*, *il-6* and *gro-b* were reported to stimulate several types of cell migration^29–35^though the role of these factors play in chondrocytes migration still remains elusive. We also found that *trpa1*, *il-1α*, *il-6* and *gro-b* can significantly exacerbate the ECM catabolism of cartilage explant or intervertebral disc^36–40^which might be helpful in improving chondrocytes migration to the edge of cartilage graft and promoting cartilage repairing in this study. Additionally, *il-8* was reported to induce chondrocyte hypertrophic differentiation and upregulate chondrogenic transcription^41,42^.

Effects of down-regulated DEGs in minced cartilage on inflammation, cell migration and matrix degradation were also investigated. Previous study^43^ found that activation of *rasl11b* can inhibit the proliferation, invasion and migration of tumor cells. *gdf6* can alleviate inflammation and degeneration of intervertebral disc^44^. Proliferation and migration of cancer cells can be reduced by *nptx1*^45^. Although functions of these down-regulated DEGs differ across studies, these results still render us to speculated that inhibition these down-regulated DEGs in CFG may promote cartilage repairing effect of minced cartilage transplantation.

It is worth noting that excessive or long-lasting inflammatory response will undoubtedly exert adverse effects on the proliferation, chondrogenic differentiation and migration of chondrocytes^46,47^. In this study, we found that the inflammation was promoted by cartilage fragmentation at mid-stage (4 and 6 WPI), and the minced cartilage-treated group had similar level of immunoreactions with chunk cartilage-treated group at 8 WPI. Coincidently, previous study proved that the speed of rat cartilage repair peaks at 4–6 weeks after injury^48^. These evidences suggest that the increased inflammatory response at peak stage of cartilage repair not only probably activate chondrocytes, but avoid persistent inflammation to induced chondrocytes apoptosis or pyroptosis to impair cartilage formation^49,50^. Therefore, exploring the optimal level of inflammatory cytokines may be helpful to find new therapeutic means to promote repairing function of cartilage transplantation.

There are some limitations in this study: (1) In this work, we only proved that minced cartilage exhibit promoted cartilage-repairing effect and immune response. More in-depth mechanistic investigations (e.g., inflammatory cytokines inhibition or genetically modified animals) are needed to elucidate the causal relationship between increased inflammation and pro-chondrogenic effect of cartilage fragmentation. In addition, deeper dive into upstream regulatory elements, transcription factors, and gene-gene interaction networks could provide broader biological insights and help identify key regulatory hubs or therapeutic targets. Future studies will prioritize validation (qPCR or Westen blotting) of top DEGs (e.g., *trpa1*, *il-8*, *il-1α*). In addition, since sample size used in this study (*n* = 3 per group/timepoint) was small, future work with expanded cohorts and longer observation period could draw more robust conclusion. (2) Only 2 sizes of cartilage fragments (0.5 mm and 2 mm) were tested in this study and whether smaller cartilage fragment can further improve cartilage repairing effect still remains undetermined. Since no obvious difference result was found between chondrocyte implantation and osteochondral transplantation^51^we suppose that there must be an optimal size of the transplanted cartilage fragments. Incorporating a broader range of fragment sizes or conducting a size optimization experiment would strengthen the translational value of this study, which warrant further exploration. (3) The key DEG that regulate the pro-chondrogenic function of particulated cartilage and related mechanism should be elaborated in future. (4) Considering the low density of chondrocytes in cartilage and limited quantity of harvestable cartilage in rats, rabbits were used to obtain cartilage for RNA-seq in this experiment since rabbits can provide enough articular cartilage and higher concentration of cartilage RNA. Although rat and rabbit are both mammal and share high genetic similarity, RNA-seq derived from rat cartilage can avoid species specificity-caused limitation. (5) While the animal model employed in this study does not fully recapitulate the multiaxial mechanical loading of the human knee joint, this non-weight-bearing site was strategically selected owing to experimental control and established preclinical protocols^52,53^. Future work should employ large-animal models (e.g., load-bearing sites in minipig) to simulate biomechanical stimuli.

## Conclusion

To sum up, this work confirmed the effectiveness of cartilage fragment transplantation on knee articular cartilage injury. We also preliminarily analyzed the potential mechanism by RNA sequencing, and found that inflammation was involved in the pro-chondrogenic effect of cartilage fragmentation. Our findings provide scientific basis for further research on particulated cartilage promoting cartilage repair in future.

## Methods

### Animals and treatments

Protocols of animal care and experiment were approved by the Ethical Committee of the First Affiliated Hospital of University of South China, and in accordance with the “Guiding Directives for Human Treatment of Laboratory Animals” issued by the Ministry of science and Technology of Peoples Republic of China. All methods are reported in accordance with ARRIVE guidelines. Animals used in this study were purchased from Hunan SJA Laboratory Animal Co., Ltd.

10 healthy male Sprague-Dawley (SD) rats (8–10 weeks, 350–500 g) were selected for cartilage donors, which were employed to harvest allogeneic cartilage from femoral condyle, tibial plateau, patella articular surface and femoral head cartilage cap after euthanasia by intraperitoneal injection of pentobarbital sodium (200 mg/kg body weight). 27 adult healthy male Sprague-Dawley (SD) rats (8–10 weeks, 350–500 g) were served as cartilage recipient animal. After anesthesia by intraperitoneal injection. midline incision (about 2 cm) on these rats` knee joints were made vertically, and exposed the femoral trochlea through the approach of the inner side of the patella. Then a circular osteochondral defect (~ 2 mm diameter and ~ 1 mm depth) was made on the femoral intercondylar fossa with a hand drill (equipped with a 2-mm drill bit). All the procedures were performed aseptically.

27 cartilage recipient rats were divided into 3 groups. Rats in Group A received minced allograft cartilage fragments (< 0.5 mm) with fibrin glue (Bioseal Biotech, Guang Zhou) implantation, while the cartilage defect area of rats in Group B were transplanted with chunk cartilage fragments (approximately 2 mm) with fibrin glue. For Group C rats, only 0.1 ml fibrin glue were used to fill the cartilage defect area. After operation, rats were free to move. At 4-, 6-, and 8-week post operation, 3 rats in each group were euthanized and the knee joint specimens were collected for following experiments.

### Gross evaluation

According to ICRS macroscopic measurement^54^each specimen was scored by 2 independent observers and then averaged to obtain the final score. The ICRS evaluation was carried out from 3 dimensions: degree of defect repair, integration to border zone, and macroscopic appearance (shown in Table 1), and full score of each dimension is 4 points. The overall repairing effect was assessed by total scores, which are graded as follows: 12 points (completely normal), 8 to 11 points (basically normal), 4 to 7 points (abnormal), and 1 to 3 points (severely abnormal).

**Table 1:** ICRS macroscopic measurement for cartilage repair.

Dimensions	Points
Degree of lesion repair (% repair of lesion depth)	0% (0), 25% (1), 50% (2), 75% (3), 100% (4)
Bonding to adjacent cartilage	0–25% of graft bonding to adjacent cartilage (0), half of graft bonding to adjacent cartilage while another half of graft with border > 1 mm (1), 75% of graft bonding to adjacent cartilage while 25% of graft with border > 1 mm (2), border < 1 mm (3), totally bonding to adjacent cartilage (4)
Macroscopic appearance	Totally degenerated area (0), several small or a few large fissures (1), small and scattered fissures (2), fibrillated surface (3), totally smooth surface (4)

## Histological assessment

The collected samples were fixed in 4% paraformaldehyde for 24 h and decalcified with 0.5 M EDTA (pH = 7.4) solution for 2 weeks. The decalcified samples were subjected to dehydration, paraffin embedding and then sliced into 5-µm sections. For each sample, 3 coronal sections were made at: middle of cartilage defect, 500 μm anterior to middle of cartilage defect, and 500 μm posterior to middle of cartilage defect. Sections were used to perform hematoxylin and eosin (HE) staining, safranin O/fast green staining, and immunohistochemical staining for collagen type II (Col-2) and interleukin-6 (IL-6), respectively. Each section was measured independently and then averaged to obtain final value of this specimen.

HE staining was carried out by using a commercial kit (Servicebio, G1076), according to manufacturer`s instruction. Briefly, after deparaffinization in xylene and rehydration in a series of ethanol, slices were subjected to hematoxylin solution for 3 min and eosin solution for 30 s. For safranin O/fast green staining, deparaffined and rehydrated slices were placed in hematoxylin solution for 2 min and then washed in running tap water for 5 min. After 0.1% safranin O (Servicebio, G1053) staining for 3 min, and 1% acetic acid rinsing for 10–15 s, slices were stained with 0.1% fast green solution (Servicebio, G1053) for 5 min, followed by washing in running tap water for 5 min. For both HE staining and safranin O staining, dehydrated sections were coverslipped with Permount and images were obtained with an Olympus CX31 optical microscope.

As described previously^55^images of HE and safranin O/fast green staining were used to evaluate histological quantification of osteochondral lesions by modified O’Driscoll scoring, which is based on hyaline content of repaired tissue, structural characteristics, degenerative changes in repaired tissue and adjacent cartilage, reconstitution of subchondral bone, bonding of repaired cartilage to subchondral bone and safranin O staining. 2 judgers evaluate each specimen independently and the final score is average score of the 2 judgers. The detail of modified O’Driscoll scoring system is shown in Table 2.

**Table 2:** Modified o’driscoll scoring for cartilage repairing evaluation.

Dimensions	Points
% Hyaline in repaired tissue	≤ 20% (0), 21–40% (2), 41–60% (4), 61–80% (6), > 80% (8)
Structural characteristics	
Surface irregularity	Severe degeneration (0), fissures (1), smooth and intact (2)
Structural integrity	Severe lack of integration (0), slight disruption including cysts (1), normal (2)
Thickness	≤ 50% (0), 51–99% (1), 100% (2) of normal adjacent cartilage
Integration to surrounding cartilage	Not integration (0), integrated at 1 end/partially at both ends (1), integrated at both ends of graft (2)
Cellular degeneration in repaired tissue (degreed of hypocellularity)	> 25% chondrocyte clusters (0); ≤25% clusters (1); no clusters (2)
Degeneration in adjacent tissue	Severe hypocellularity, slight staining (0); mild hypocellularity, slight staining (1); normal cellularity, mild clusters, moderate staining (2); normal cellularity, no clusters, normal staining (3)
Reconstitution of subchondral bone	≤ 50% (0), 51–100% (1), complete reconstitution (2)
Integration of repaired cartilage to *de novo* subchondral bone	≤ 50% (0), 51–100% (1), complete and uninterrupted (2)
Safranin O staining	≤ 40% (0), 41–80% (1), > 80% (2)

Immunohistochemical analyses were conducted as described previously^56^. Sections were incubated with primary antibodies against Col-2 (Servicebio, GB12021, 1:1000) and IL-6 (Servicebio, GB11117, 1:200), respectively. Secondary antibody (GB23302, 1:500) was also purchased from Servicebio, in accordance with manufacturer`s instruction. The average optical densities were verified in three sections per rat and three rats per group by using Image J software (version 1.51).

### ***Ex vivo*culture of rabbit cartilage**

RNA-seq was performed in rabbits to overcome technical limitations in RNA yield from rat cartilage. After general anesthesia, total knee cartilage was obtained from 4-week-old male rabbits aseptically, and divided into 2 groups according to different cartilage preparations: CFG (cartilage fragment group, cut cartilage into about 0.5 × 0.5 × 0.5 mm fragments) and CCG (cartilage chunk group, 2 mm-diameter and 1 mm-thick cartilage blocks). Then cartilage fragments or cartilage chunk were evenly seeded on gelatin sponge (1 × 1 × 1 cm), and the fibrin glue was added. After solidification, the mixture was cut into blocks in a size of about 5 × 5 × 2 mm. The explants were cultured in 6-well plate at 37 ℃ in an atmosphere containing 5% CO_2_ in DMEM (Dulbecco’s Modified Eagle Medium) containing 10% fetal bovine serum. After 4-week culture, samples were sent for following RNA-seq.

## RNA isolation

Total cartilage RNA was extracted according to Trizol’s (Invitrogen, 15596018CN) instructions. Samples were ground in liquid nitrogen and transferred to 2 ml tubes containing 1.5 ml Trizol reagent. The mixture was centrifuge at 12,000 × g for 5 min at 4 ℃. Then, the supernatant was transferred to a new 2.0 ml tube and added with 0.3 ml of chloroform/isoamyl alcohol (24:1) per 1.5 ml of Trizol reagent. After centrifugation at 12,000 × g for 10 min at 4 °C, the aqueous phase of the mixture was transferred to a new 1.5 ml tube with equal volume of supernatant of isopropyl alcohol, and supernatant was removed after centrifugation for 12,000 × g for 20 min at 4 ℃. After washing with 1 ml 75% ethanol, the RNA pellet was air-dried and then dissolved by adding 100 µL of DEPC-treated water.

### Long non-coding RNA library construction

Nano drop and Agilent 2100 bioanalyzer (Thermo Fisher Scientific) were used to qualified and quantified the total RNA. Ribo-Zero™ Magnetic Kit (Epicentre) is used to remove rRNA. The retrieved RNA was fragmented by adding First Strand Master Mix (Invitrogen). Then the pre-prepared first-strand synthesis reaction mixture is added to the fragmented RNA, and the reaction system and program are configured and set up for second-strand cDNA synthesis, using dUTP instead of deoxythymidine triphosphate (dTTP). The synthesized cDNA was subjected to terminal repair and then 3’adenylate acidification. Adapters were attached to the ends of these 3’adenylate cDNA fragments. Several rounds of PCR amplification were performed with PCR Primer Cocktail and PCR Master Mix to enrich cDNA fragments. Then the PCR products were purified with Ampure XP Beads. The final library was qualified and quantified by two methods: the distribution of fragment size was examined by Agilent 2100 Bioanalyzer, and the library was quantified by real-time quantitative PCR (qPCR) (TaqMan probe).

### Sequencing

The sequencing data was filtered with SOAPnuke (v1.5.2) by (1) Removing reads containing sequencing adapter; (2) Removing reads whose low-quality base ratio (base quality less than or equal to 15) is more than 20%; (3) Removing reads whose unknown base (‘N’ base) ratio is more than 5%. Afterwards, clean reads were obtained and stored in FASTQ format. The clean reads with a Q30 value greater than 95% were mapped to the reference genome using HISAT2 (v2.0.4). After that, Ericscript (v0.5.5) and rMATS (V3.2.5) were used to detect fusion genes and differential splicing genes (DSGs), respectively. Bowtie2 (v2.2.5) was applied to align the clean reads to gene set, a database established by BGI (Shenzhen Beijing Genomics Institute), in which known and novel, coding and noncoding transcripts were included. Then the expression level of gene was calculated by RSEM (v1.2.12). The heatmap was drawn by Pheatmap (V1.0.8) according to the gene expression difference in different samples. Essentially, differential expression analysis was performed using the DEGSeq2 (v1.4.5) with Q value ≤ 0.05. To take insight into change of phenotypes, GO (www.geneontology.org) and KEGG (www.kegg.jp) enrichment analyses of annotated different expression gene were performed by Phyper (https://en.wikipedia.org/wiki/Hypergeometric_distribution) based on Hypergeometric test. The significant levels of terms and pathways were corrected by Q value with a rigorous threshold (Q value ≤ 0.05) by Bonferroni.

### Statistical analysis

Statistical analysis was performed on the data with GraphPad Prism software (version 10.2.2). All data were expressed as mean ± standard deviation (SD). Normal distribution of data was confirmed by Shapiro-Wilk test (*P* > 0.05). Two-way analysis of variance (ANOVA) with Turkey post-hoc test was used for multiple-group comparisons. For all experiments, *P* < 0.05 was considered to be significant.

## Electronic supplementary material

Below is the link to the electronic supplementary material.

Supplementary Material 1

## Data Availability

The RNA sequence data analyzed during the current study are available in the Genome Sequence Archive (GSA) repository (accession number: CRA021469).
